# Prebiotic Potential of Brewer’s Spent Grain Residual Solid After Enzymatic Hydrolysis: Evidence from a Colonic Fermentation Study

**DOI:** 10.3390/foods15132378

**Published:** 2026-07-03

**Authors:** María José Vargas-Straube, Francisca Rojas-Hidalgo, Jordana Nunes de Oliveira, Boris Arancibia, Thatyane Mariano de Albuquerque, Taliana Kênia Alencar Bezerra, Eike Guilherme Torres de Souza, Evandro Leite de Souza, María Salomé Mariotti-Celis, María Elvira Zuñiga, Lida Fuentes, Carmen Soto-Maldonado

**Affiliations:** 1 Centro Regional de Estudios en Alimentos Saludables (CREAS), Valparaíso 2373223, Chile; mvargas@creas.cl (M.J.V.-S.); francisca.rojas@creas.cl (F.R.-H.); barancibia@creas.cl (B.A.); mzuniga@ucv.cl (M.E.Z.); carmensoto@creas.cl (C.S.-M.); 2 Pontificia Universidad Católica de Valparaíso, Valparaíso 2362804, Chile; 3 Laboratory of Food Microbiology, Department of Nutrition, Federal University of Paraíba, João Pessoa 58051-900, Brazil; jordana.nunes@academico.ufpb.br (J.N.d.O.); thatyane.albuquerque3@academico.ufpb.br (T.M.d.A.); els@academico.ufpb.br (E.L.d.S.); 4 Multi-User Food Quality Laboratory, Department of Food Engineering, Federal University of Paraíba, João Pessoa 58051-900, Brazil; taliana.kenia@hotmail.com (T.K.A.B.); eikesouza77@gmail.com (E.G.T.d.S.); 5 Faculty of Medicine and Health, Universidad Finis Terrae, Santiago 7501015, Chile; mmariotti@uft.cl

**Keywords:** brewer’s spent grain, prebiotic fiber, arabinoxylans, ferulic acid, short-chain fatty acids, gut microbiota

## Abstract

Brewer’s spent grain (BSG), the main by-product of the brewing industry, is a rich source of arabinoxylans (AXs) and hydroxycinnamic acids, particularly ferulic acid (FA), which contribute to its prebiotic potential. This study evaluates the prebiotic properties of residual solid from an enzymatically treated BSG, compared to the properties of BSG as a non-enzymatically hydrolyzed control. Although the residual solid exhibited total polyphenol (2581.96 ± 70.63 mg/100 g dry weight) and FA (180.84 ± 3.28 mg/100 g dry weight) contents comparable to those of the non-hydrolyzed control (2500.38 ± 284.20 and 179.59 ± 3.30 mg/100 g dry weight, respectively), the AX content was significantly higher (14,084.81 ± 185.72 mg/100 g), accompanied by a lower degree of feruloylation (12.84 ± 0.23 mg FA/g AX), higher antioxidant activity (64,825.35 ± 4011.24 μmol TE/100 g), and structural changes visualized by scanning electron microscopy. In addition, in vitro colonic fermentation showed a delayed butyrogenic profile, with increased butyrate production compared to the control (3.17 ± 1.44 mM). Microbiota analysis by fluorescence in situ hybridization (FISH) coupled with flow cytometry indicated an increase in butyrate-producing bacteria, including *Faecalibacterium prausnitzii* (+5.90) and *Eubacterium rectale* (+6.88). Growth of *Bacteroides*, *Lactobacillus*, and *Bifidobacterium* spp. was also promoted. Overall, these findings suggest that enzymatic processing of BSG can generate a residual solid with modified structural characteristics and potential prebiotic functionality, supporting its potential application as a fermentable flour ingredient in functional foods.

## 1. Introduction

Increased agricultural production and food processing generate large quantities of lignocellulosic residues, among which brewer’s spent grain (BSG) is the most abundant agro-industrial byproduct of the brewing sector. Produced after wort extraction during beer manufacturing, BSG accounts for more than 85% of the brewing industry’s byproducts, reaching approximately 36.4 million tons annually worldwide [1]. This insoluble solid residue is rich in dietary fiber, proteins, minerals, and phenolic compounds [2,3]. Although it has traditionally been used as animal feed or soil amendment, its potential applications in human nutrition and as a source of high-value bioproducts have attracted increasing attention [4,5,6,7].

Hemicellulose represents approximately 20–40% of BSG dry matter and is mainly composed of arabinoxylans (AXs). These polysaccharides consist of a β-(1→4)-linked xylose backbone substituted with arabinose residues and are characteristically esterified with polyphenol compounds such as hydroxycinnamic acids (e.g., ferulic and p-coumaric) in cereal matrices [3,8,9,10]. FA exhibits strong antioxidant activity, making it valuable in food, cosmetic, and pharmaceutical applications [11]. Moreover, the health-promoting effects of BSG are largely attributed to the prebiotic properties of feruloylated AX, which are selectively fermented by the gut microbiota [12]. Their fermentation promotes the production of short-chain fatty acids (SCFAs) and the proliferation of beneficial intestinal bacterial groups. During this process, fiber-bound FA is released by microbial polysaccharide-degrading enzymes, including xylanases and feruloyl esterases. Among the taxa involved, species of *Bacteroides* preferentially degrade less-substituted AX structures, whereas *Bifidobacterium* and *Lactobacillus* are associated with the metabolism of more highly branched AX structures [12]. Additional contributors include members of the families *Prevotellaceae*, *Ruminococcaceae*, and *Lachnospiraceae* [13]. The fermentation of these polysaccharides primarily generates acetate, propionate, butyrate, and lactate—metabolites that contribute to intestinal homeostasis and to the metabolic benefits associated with AX-rich substrates [12,13].

Recent valorization strategies for BSG use enzymatic hydrolysis with feruloyl esterases and carbohydrases to produce AX-rich hydrolysates with potential prebiotic functionality and release FA, known for its antioxidant activity [4,6,12,14,15,16,17,18,19]. Such approaches are attractive due to their selectivity, mild operating conditions, compatibility with food systems, and reduced environmental impact. Similar to other processing strategies, including chemical hydrolysis [14,20] and physical treatments [21], the primary aim of enzymatic processing is to obtain an FA-enriched liquor followed by an AX-rich hydrolysate or extract, leaving a residue solid with prebiotic potential.

Even though few studies have addressed the valorization of the residual solid fraction obtained after processing [22,23,24], some have reported the prebiotic functionality of residual flours obtained after solid–liquid extraction and ohmic heating treatments of BSG [25]. This fraction remains structurally modified but retains dietary fiber associated with polyphenols that confer prebiotic activity [25]. In this context, the production of functional flours from pretreated BSG has recently emerged as a promising strategy for valorizing this agro-industrial by-product [26,27,28]. The implementation of a biorefinery approach enables the integral valorization of BSG, allowing the sequential recovery of bioactive compounds such as FA and the generation of functional fiber fractions with potential prebiotic applications [29].

This study evaluates the in vitro potential prebiotic effect of the residual solid obtained after the enzymatic processing of BSG using a selective, low-environmental-impact treatment that structurally modifies the hemicellulosic matrix, but maintains a high content of bioactive compounds. This modification is expected to improve the bioaccessibility of bioactive compounds and modulate fermentative responses.

## 2. Materials and Methods

### 2.1. Raw Materials and Reagents

BSG was supplied by a micro-enterprise located in the Valparaíso Region, Chile (33°02′ S, 71°37′ W). The material was obtained from a single batch of Session India Pale Ale (IPA) beer and was collected and frozen on the same day the by-product was generated. Comparable chemical composition profiles have been observed among different batches of the same beer type collected following the same procedure (Table S4).

All chemicals and reagents were of analytical grade. Sodium hydroxide (NaOH), phosphoric acid, phosphate buffer, and sodium carbonate were supplied by Loba Chemie Pvt. Ltd. (Mumbai, MH, India); acetonitrile, methanol, and ethanol were provided by Scharlab S.L (Sentmenat, CT, Spain); hydrochloric acid (HCl) was provided by Central Drug House (P) Ltd. (Delhi, DL, India). Ferulic acid, xylose, gallic acid and trifluoroacetic acid, Folin–Ciocalteu, sinapic acid, p-coumaric acid, chlorogenic acid, caffeic acid, 2,2′-azobis(2-amidinopropane) dihydrochloride (AAPH), fluorescein, Trolox, sodium chloride (NaCl), potassium chloride (KCl), sodium bicarbonate (NaHCO_3_), magnesium sulfate (MgSO_4_), calcium chloride (CaCl_2_), ferrous sulfate (FeSO_4_), potassium dihydrogen phosphate (KH_2_PO_4_), phosphate-buffered saline (PBS), Tween 80, resazurin fructooligo-saccharides (FOS) and analytical standards of individually packaged organic acids (propionic, acetic, butyric, lactic, citric, tartaric, malic, and succinic acids ) included in the Organic Acids Kit were provided Merck KGaA (Darmstadt, HE, Germany). SYBR Green staining was provided by Molecular Probes (Invitrogen, Carlsbad, CA, USA). FISH oligonucleotide probes were synthesized by Metabion International AG (Planegg, Bavaria, Germany). Enzymes (α-amylase and pancreatin) and bile salts were obtained from Merck KGaA (Darmstadt, Germany). Pepsin was supplied by Dinâmica Química (Brazil).

### 2.2. Enzymatic Hydrolysis of BSG

BSG was stabilized and enzymatically pretreated according to protocols previously established by our group. The reproducibility of the hydrolytic process, in terms of arabinoxylan (AX) solubilization across different batches of the same IPA beer type, was confirmed (Table S5). BSG was stabilized by convective drying at 60 °C for 21 h using a Heat Pump Food Dehydrator (AGHD-15ELC, AIM Energy Saving Technology Co., Ltd., Zhongshan, China), and the particle size was adjusted to 1 mm using an electric mill (MF 10 basic, IKA-Werke GmbH & Co. KG, Staufen, Germany). The stabilized BSG was then hydrolyzed with a commercial β-glucanase under the supplier’s conditions (Ultraflo^®^ L, Novozymes, Bagsværd, Denmark), which has been reported to release FA from BSG [30,31]. After enzymatic hydrolysis, the system was separated into a liquid fraction (hereafter referred to as hydrolyzed liquid fraction, HLF) and a residual solid fraction (hereafter referred to as hydrolyzed residual solid fraction, HRSF). The residual solid was freeze-dried using a dual-condenser benchtop lyophilizer (Freeze Dryer, FDT-8632, OPERON Co., Ltd., Gimpo-si, Republic of Korea) at −25 ± 2 °C. Two biological replicates were performed for each fraction. The stabilized BSG, after being convectively dried and milled to a particle size of 1 mm, was used as an unhydrolyzed control (hereafter referred to as the non-hydrolyzed brewer’s spent grain, NHBSG).

### 2.3. Characterization of BSG Samples

#### 2.3.1. Scanning Electron Microscopy (SEM) Analysis

Solid samples from BSG (HRSF and NHBSG) were morphologically analyzed via SEM. Samples were mounted on 12.7 mm diameter specimen mounts (Ted Pella, Inc., Redding, CA, USA) pre-coated with 3M conductive copper tape to ensure proper conductivity and sample adhesion. To prevent charging artifacts and improve image quality, samples were sputter-coated with a 2 nm thick gold–palladium (Au-Pd) layer using a Leica EM ACE 200 (Leica Microsystems, Wetzlar, Germany) high vacuum coating system under standard operating conditions. Morphological analysis was performed using an AURIGA Compact dual-beam microscope (Carl Zeiss, Oberkochen, Germany) equipped with field emission electron microscopy (FESEM), scanning transmission electron microscopy (STEM), and focused ion beam (FIB) capabilities. Images were acquired at magnifications ranging from 200× to 10,000× under optimized conditions. Four independent sections per sample were analyzed.

#### 2.3.2. Differential Thermogravimetric Analysis (TGA)

Solid samples HRSF and NHBSG were thermally characterized by TGA. Samples (10.0 ± 0.5 mg) were placed in platinum pans and heated from 25 to 900 °C at a rate of 10 °C·min^−1^ in a DTG-60H (Shimadzu, Kyoto, Japan). Measurements were carried out at atmospheric pressure under a nitrogen flow of 100 mL·min^−1^ (99.90% purity). Small particle size (1 mm), low sample mass, and the selected heating rate were used to minimize heat- and mass-transfer limitations, following recommended TGA conditions adapted from Marchese et al. (2024) [32]. In accordance with ICTAC Kinetics Committee guidelines [33], the TGA cell was purged with nitrogen before each run to ensure an oxygen-free atmosphere and prevent sample oxidation. Each heating program was repeated at least three times to confirm repeatability. Raw TGA data were processed according to El-Sayed et al. (2024) [34], to determine mass percentage (TG%) relative to initial sample weight and derivative thermogravimetric (DTG) values expressed as mass loss rate (mg/min) through numerical time-based differentiation, with analysis restricted to the 50–650 °C temperature range and DTG values within 0–2 mg/min to exclude artifacts.

#### 2.3.3. Proximate Composition Analysis

Proximate composition was determined for samples obtained after enzymatic hydrolysis (HLF and HRSF), as well as NHBSG, according to standard methods of AOAC International [35]. Moisture content was measured by oven drying at 105 °C until constant weight (AOAC 925.10), ash content by incineration at 500 °C (AOAC 923.03), protein content by digestion and distillation using the Kjeldahl method (AOAC 960.52), and crude fat by solvent extraction using the Soxhlet method (INN, 1988). Dietary fiber was determined using an enzymatic–gravimetric method (K-TDFR; Megazyme Ltd., Bray, Co., Wicklow, Ireland), and carbohydrate content was calculated by difference. All analyses were performed in triplicate.

#### 2.3.4. Determination of Hydroxycinnamic Acid Profile

The hydroxycinnamic acid profile (chlorogenic, caffeic, p-coumaric, sinapic, and ferulic acids) was determined by HPLC-DAD (Jasco MD-4015, JASCO Corporation, Tokyo, Japan) according to a method adapted from Kareparamban et al. (2013) [36] and Nadal et al. (2015) [37] in NHBSG, HLF and HRSF. For NHBSG and HRSF, samples were subjected to alkaline hydrolysis prior to analysis. Briefly, 1 g of sample was subjected to alkaline hydrolysis with 20 mL of 4% *w*/*v* NaOH for 1 h at 60 °C and 120 rpm, followed by neutralization to a pH of 6–7, filtration, and HPLC analysis. HLF was analyzed directly without alkaline hydrolysis, since it represents the soluble fraction obtained after enzymatic hydrolysis of NHBSG.

Chromatographic separation was performed using a Kromasil C18 column (250 × 4.6 mm, 5 μm) with a mobile phase consisting of 0.1 N aqueous phosphoric acid, acetonitrile, and methanol (75:15:10, *v*/*v*/*v*) under isocratic conditions. The flow rate was maintained at 1.0 mL/min for 18 min, with the column temperature set at 25 °C. Detection was carried out at 320 nm. Quantification was achieved using external calibration curves (1–1000 mg/L; R^2^ > 0.9995) prepared for ferulic, sinapic, p-coumaric, chlorogenic, and caffeic acids. The analytical validation parameters are indicated in Table S1. Before analysis, sample extracts were filtered through 0.22 μm membrane filters and injected at a volume of 10 μL. All samples were analyzed in triplicate. Data acquisition and analysis were performed using ChromNAV 2.0 software. Results were expressed as mg hydroxycinnamic acid per 100 g of dry sample. For NHBSG and HRSF, values were calculated on dry weight basis, whereas for HLF, results were reported as the amount of hydroxycinnamic acids recovered per 100 g of BSG subjected to enzymatic hydrolysis.

#### 2.3.5. Determination of In Vitro Antioxidant Activity

In vitro antioxidant activity of alkaline HRSF and NHBSG hydrolysates was assessed using the oxygen radical absorbance capacity (ORAC) assay according to Prior et al. (2005) [38], with modifications described by Valdenegro et al. (2021) [39]. AAPH was used as the peroxyl radical generator and fluorescein as the oxidizable probe. Briefly, 25 μL of the sample diluted in 75 mM phosphate buffer (pH of 7.4) was mixed with 150 μL of fluorescein solution in a 96-well microplate. Subsequently, 25 μL of AAPH solution (271.2 g/mol) was added to initiate the reaction at 37 °C. Fluorescence decay was monitored every minute for 60 min at 485 nm excitation and 538 nm emission using a Fluoroskan Ascent Multi-Mode Microplate Reader (Fluoroskan Ascent, Thermo Scientific, Waltham, MA, USA), with orbital shaking before each reading. Antioxidant capacity was quantified using the area under the fluorescence decay curve and calculated from a quadratic regression equation derived from a Trolox standard curve. Results were expressed as μmol Trolox equivalents (TE) per 100 g on a dry weight basis. All analyses were performed in triplicate.

#### 2.3.6. Determination of Arabinoxylan Content and Feruloylation Degree

Given the focus of this study on the prebiotic potential of HRSF, both HRSF and its control, NHBSG, were further characterized by determining the AX content. AX content was quantified following acid hydrolysis using the orcinol–HCl colorimetric method for pentoses [40]. Briefly, 20 mg of the dried sample was hydrolyzed with 2 mL of 2 mol/L trifluoroacetic acid at 105 °C overnight. After cooling to room temperature, the hydrolysate was centrifuged (5000× *g*, 10 min), and the supernatant was collected. An aliquot (2 mL) of the hydrolysate was mixed with 2 mL of orcinol –HCl reagent (0.1% *w*/*v* orcinol in concentrated HCl containing 0.1% *w*/v ferric chloride). The mixture was heated at 100 °C for 30 min and cooled to room temperature, and the absorbance was measured at 580 nm and 670 nm. The absorbance difference (A_670_ − A_580_) was used for quantification. Xylose was used as the calibration standard (5–200 mg/L). Results were expressed as mg AX per 100 g on a dry weight basis. All analyses were performed in triplicate.

The degree of feruloylation was calculated as the ratio of FA content, determined by HPLC after alkaline hydrolysis, to AX content and expressed as mg FA/g AX, according to Carvajal-Millan et al. (2005) [41].

In addition, soluble arabinoxylan-derived carbohydrates in HLF were estimated using the orcinol–HCl colorimetric assay. Unlike solid fractions, no prior acid hydrolysis was applied, since the HLF already contains water-soluble carbohydrate species derived from arabinoxylan degradation. This approach reflects the soluble fraction of hemicellulose-derived carbohydrates released during enzymatic hydrolysis. Results were expressed as mg AX per 100 g of dry BSG subjected to enzymatic hydrolysis. All analyses were performed in triplicate.

#### 2.3.7. Determination of Total and Soluble Polyphenol Content

Total phenolic content (TPC) of HRSF and NHBSG was determined following alkaline hydrolysis using the Folin–Ciocalteu assay [42]. Briefly, 1 g of sample was subjected to alkaline hydrolysis as described above. Subsequently, 0.5 mL of appropriately diluted sample was mixed with 0.25 mL of 50% *v*/*v* Folin–Ciocalteu reagent and 3.75 mL of distilled water. Then, 0.5 mL of 10% *w*/*v* sodium carbonate solution was added. The mixture was incubated in the dark at room temperature for 1 h, and absorbance was measured at 765 nm. Gallic acid was used for calibration (2.5–150 mg/L). Results were expressed as mg gallic acid equivalents (GAE) per 100 g on a dry weight basis [42]. All analyses were performed in triplicate.

Finally, in addition to the determination of total phenolic compounds, including FA, in alkaline HRSF and NHBSG hydrolysates, soluble (non-covalently bound) phenolic compounds were also determined in hydroalcoholic extracts of these samples. Soluble phenolic compounds were extracted from 1 g of sample using 20 mL of 80% *v*/*v* ethanol for 1 h at 60 °C and 120 rpm, followed by centrifugation (5000× *g*, 10 min). TPC and FA in the hydroalcoholic extracts were quantified following the same procedures described above for the alkaline hydrolysates. All analyses were performed in triplicate.

### 2.4. In Vitro Gastrointestinal Digestion

In vitro gastrointestinal digestion was performed using the standardized INFOGEST protocol described by Minekus et al. (2014) [43], with minor modifications. These modifications, including enzyme activities and post-digestion dialysis conditions, were applied as described in Silva et al. (2025) [44]. Briefly, 10 g of BSG solid samples (HRSF and NHBSG) were subjected to simulated oral, gastric, and intestinal phases using specific digestive fluids and enzymes (α-amylase, 75 U/mL; pepsin, 2000 U/mL; pancreatin, 100 U/mL). The pH of each phase was adjusted using 6 mol/L HCl or 5 mol/L NaOH. Digestion was carried out at 37 °C in a shaking incubator (SP-222, SPLABOR, São Paulo, Brazil) to mimic peristaltic movements. After digestion, samples were subjected to a dialysis step using regenerated cellulose membranes (1 kDa cutoff; Spectra/Por 6, Spectrum Europe B.V., Breda, The Netherlands). Membranes were immersed in 0.01 mol/L NaCl and incubated at 5 ± 0.5 °C for 18 h. This step was implemented to operationally separate low-molecular-weight, dialyzable compounds—representing the fraction potentially available for passive intestinal diffusion—from the high-molecular-weight, non-dialyzable fraction, which was subsequently used as substrate for in vitro colonic fermentation. This approach has been previously applied in in vitro digestion systems to approximate intestinal bioaccessibility and passive diffusion of phenolic compounds [44]. Importantly, the dialysis step does not aim to fully reproduce in vivo intestinal absorption, but rather to provide a functional partition between potentially bioaccessible and non-bioaccessible fractions. While it is acknowledged that the removal of low-molecular-weight compounds may reduce the pool of substrates potentially available for colonic fermentation in vivo, this methodological step is inherent to coupled digestion–fermentation models that seek to differentiate systemic bioaccessibility from colonic substrate availability [44]. Therefore, the non-dialyzable fraction is interpreted as the fraction remaining after simulated intestinal passage under the applied in vitro conditions. The resulting materials were stored at 5 ± 0.5 °C until further use [45]. The same procedure was applied to an FA solution (0.01 g/L), following Lu et al. (2024) [46], and the resulting system was used as a control for feruloylated samples in the in vitro colonic fermentation assay. Two biological replicates were performed for each sample.

### 2.5. Preparation of Human Fecal Inoculum

The in vitro colonic fermentation procedure was conducted after approval by an Ethics Committee on Research with Human Subjects (Health Science Center, Federal University of Paraíba, Joao Pessoa, PB, Brazil; protocol number 6,259,560, CAAE: 71081023.1.0000.5188) due to the use of human feces. Before participating in the research, fecal donors signed an Informed Consent Form (ICF), consenting to the use of their material in the experiment. Eight healthy adult volunteers (four men and four women, aged 20–39 years) donated fresh fecal samples. Donors reported no history of colonic diseases, omnivorous diet, and no use of concentrated probiotic and prebiotic foods, antibiotics, or medications in the past six months.

The fecal samples from each donor were collected in sterile tubes and placed in an anaerobic jar with an anaerobic generation system (AnaeroGen, Basingstoke, UK). Fresh samples, used 3 h after collection, were mixed in equal amounts (1:1:1:1:1:1:1:1, *w*/*w*), diluted (1:10 *w*/*v*), and homogenized (200 rpm, 2 min) in sterile phosphate-buffered saline (0.1 mol/L PBS; pH of 7.4). The mixture was filtered through a sterile triple-layer gauze and stored with 20% *v*/*v* of glycerol at −20 °C. The use of a pooled fecal inoculum enabled the comparison of the hydrolyzed (HRSF) and non-hydrolyzed (NHBSG) brewer’s spent grain samples while minimizing inter-individual variability. Although this approach masks donor-specific microbial responses, it provides a more representative assessment of the effects of the fermentable substrates on potential gut microbiota modulation by reducing the influence of individual microbiota composition [47].

### 2.6. In Vitro Colonic Fermentation System

The in vitro colonic fermentation system consisted of 40% *v*/*v* culture medium containing 4.5 g NaCl, 4.5 g KCl, 1.5 g NaHCO_3_, 0.69 g MgSO_4_, 0.8 g L-cysteine, 0.5 g KH_2_PO_4_, 0.4 g bile salts, 0.08 g CaCl_2_, 0.005 g FeSO_4_, 1 mL Tween 80, and 4 mL resazurin solution (0.25 g/L), combined with 40% *v*/*v* fecal inoculum and 20% *w*/*v* dialyzed samples obtained from complete in vitro gastrointestinal digestion. Fermentation controls were prepared, according to Albuquerque et al. (2021) [45] and Stewart et al. (2008) [48], using the same system described above, in which the 20% *w*/*v* dialyzed samples were replaced with fructooligosaccharides at 10 g/L (FOS, positive control) or water (NC, negative control without fermentable substrate), according to Silva et al. (2025) [44]. The fermentation system was adjusted to a pH of 6.8 using 1 mol/L NaOH to simulate colonic conditions. Fermentation was carried out at 37 ± 1 °C for 48 h under anaerobic conditions using an AnaeroGen system. Two biological replicates were performed for each sample.

### 2.7. Determination of Intestinal Microbial Metabolic Activity During In Vitro Colonic Fermentation

The metabolic activity of intestinal microorganisms present in the fermentation media was evaluated at 0, 24, and 48 h of in vitro colonic fermentation. For this purpose, the levels of short-chain fatty acids (SCFAs) (propionic, acetic, and butyric acid) and organic acids (citric, tartaric, malic, succinic, and lactic acid) were quantified by HPLC. The analysis was performed with an Agilent chromatograph (model 1260 Infinity LC, Agilent Technologies, St. Clara, CA, USA) with a quaternary solvent pump (G1311Cmodel), degasser, thermostatic column compartment (G1316A model), and automatic auto-sampler (G1329B model), coupled with a diode array detector (DAD) (DEGEB00715 model) and refractive index detector (RID) (G1362A model). Analytical conditions were an Agilent Hi-Plex H column (7.7 mm × 300 mm, 8 μ), a mobile phase of 0.009 mol/L H_2_SO_4_ in ultrapure water, and a flow rate of 0.6 mL/min. Data was processed with OpenLAB CDS ChemStation Edition software (Agilent Technologies). HPLC sample peaks were identified by comparing their retention times with those of organic acid standards (Sigma-Aldrich). Average peak areas were used for quantification [45]. Results were expressed as individual SCFA concentrations and net concentration changes over time. Net concentration changes were calculated as the mean concentration change relative to T_0_ (Δ concentration = concentration_t − concentration_T_0_) for each fermentation substrate × SCFA combination at 24 h and 48 h. All analyses were performed in duplicate biological replicates, each measured in two to three technical replicates.

### 2.8. Determination of the Relative Abundance of Intestinal Bacterial Groups During In Vitro Colonic Fermentation

The relative abundance of selected human intestinal bacterial groups was determined at 0, 24, and 48 h of in vitro colonic fermentation system using the fluorescence in situ hybridization (FISH) method with oligonucleotide probes targeting specific regions of the 16S rRNA gene, coupled with flow cytometry (FC). A total of eleven group-specific probes were used: Lab158 (*Lactobacillus* spp./*Enterococcus* spp.), Bif164 (*Bifidobacterium* spp.), Rfla729 (*Ruminococcus albus*/*R. flavefaciens*), Muc1437 (*Akkermansia muciniphila*), Erec482 (*Eubacterium rectale*/*Clostridium coccoides*), Rint623 (*Roseburia intestinalis*/*R. cecicola*), Fpra655 (*Faecalibacterium prausnitzii* A2-165 and L2-6), Bac303 (*Bacteroides* spp./*Prevotella* spp.), Chis150 (*Clostridium histolyticum*), Ent183 (Enterobacteriaceae), and FusAll307 (*Fusobacterium* spp.). The total bacterial population was quantified using SYBR Green staining. At 0, 24, and 48 h of fermentation, 1.5 mL aliquots were collected from each fermentation vessel, fixed overnight in 4% *v*/*v* paraformaldehyde at 4 ± 0.5 °C, and subsequently hybridized with the respective fluorescent probes according to the protocol described by Albuquerque et al. (2021) [45]. Enumeration of hybridized cells was performed using a BD Accuri C6 flow cytometer (BD Biosciences, East Rutherford, NJ, USA) equipped with a 488 nm blue solid-state laser. Fluorescence signals from individual cells were recorded as cytograms using BD Accuri C6 software (BD Biosciences). Each fermentation condition was analyzed in biological duplicate (*n* = 2). Results were calculated as the relative abundance (RA%) of cells hybridized with each specific probe group relative to the total bacterial population stained with SYBR Green [49] and expressed as compositional percentages by normalizing each group’s RA% to the sum of all eleven probe-positive fractions, thereby reflecting shifts in community composition independently of total bacterial density. Additionally, data were expressed as fold change (FC) in RA at 24 and 48 h relative to baseline (0 h) values, calculated per replicate as log2 FC (RA 24 h/% RA 0 h) and log2 FC (% RA 48 h/% RA 0 h). All metrics were summarized as the mean and range of the two replicates. No inferential statistical tests were applied owing to the absence of triplication.

### 2.9. Statistical Analysis and Data Visualization

All experiments were performed in biological duplicate (independent experiments), and each measurement was conducted in triplicate (technical replicates). For each biological replicate, data was expressed as the mean ± standard deviation of the technical replicates (*n* = 3).

For chemical data, statistical analyses were performed using Student’s *t*-test or one-way analysis of variance (ANOVA), followed by Tukey’s post hoc test. For fermentation data, statistical analyses were performed using the Kruskal–Wallis test, followed by Dunn’s multiple-comparison post hoc test with Benjamini–Hochberg (BH) correction. Differences were considered statistically significant at *p* < 0.05.

Data analysis and visualization were performed using JASP (v0.19.7; JASP Team, 2026) and R (v4.6.0; R Core Team, 2026 [50]). The following R packages were used: dplyr, ggalluvial, rstatix, ggplot2, patchwork, cowplot, multcompView, and openxlsx.

## 3. Results and Discussion

### 3.1. Chemical and Structural Characterization of BSG Samples

The proximate analysis showed that the NHBSG used in this study contained 19.68 g/100 g available carbohydrates and a high total dietary fiber (TDF) content (48.67 g/100 g), mainly insoluble dietary fiber (IDF, 46.08 ± 0.00 g/100 g) (Table 1), exhibiting the typical chemical characteristics generally reported for this agro-industrial residue [7]. Following enzymatic hydrolysis with commercial β-glucanase, HRSF was predominantly composed of dietary fiber, proteins, and total carbohydrates, comparable to NHBSG. However, HRSF showed lower protein (18.37 ± 0.14 g/100 g vs. 19.85 ± 0.23 g/100 g), IDF (41.71 ± 0.00 g/100 g vs. 46.08 ± 0.00 g/100 g), and SDF (1.66 ± 0.00 vs. 2.59 ± 0.00 g/100 g) contents than NHBSG, while total carbohydrates were higher (11.98 ± 0.13 g/100 g vs. 7.91 ± 0.10 g/100 g). In the case of HLF, solubilization of carbohydrates (0.50 ± 0.08 g/100 g) and fiber (0.71 g/100 g TDF) was observed. Also, 0.20 ± 0.01 g/100 g of ether extract and 0.14 ± 0.01 g/100 g of protein were observed. Overall, these results indicate that enzymatic hydrolysis partially solubilized carbohydrates while retaining most of the dietary fiber in the residual solid fraction.

Regarding hydroxycinnamic acid content, HRSF and NHBSG showed similar levels of FA (180.84 ± 3.28 vs. 179.59 ± 3.30 mg/100 g), sinapic acid (498.29 ± 16.32 vs. 472.83 ± 54.78 mg/100 g), and p-coumaric acid (3.53 ± 0.14 vs. 2.88 ± 0.60 mg/100 g). For their part, in HLF were recovered 43.95 ± 2.22 mg of FA and 1.57 ± 1.28 mg of sinapic acid per 100 g of BSG subjected to enzymatic hydrolysis. Neither chlorogenic acid nor caffeic acid was detected in any BSG fraction (Table S2). It is worth noting that the FA content determined in NHBSG by alkaline hydrolysis is consistent with values previously reported in other studies, ranging from 46 to 291 mg/100 g [2,8,20,51,52]. On the other hand, the FA content determined in HLF is consistent with the expected yields obtained after carbohydrate hydrolase treatments [30,31], since these enzymes hydrolyze β-glucans and hemicellulose, exposing the fiber matrix without fully releasing the total FA content [2]. Consequently, one of the advantages of partial FA solubilization after enzymatic processing is the production of an HRSF with potential bioactive properties that, together with its high dietary fiber content, may exert a potential prebiotic effect following colonic fermentation [53].

**Figure 1:**
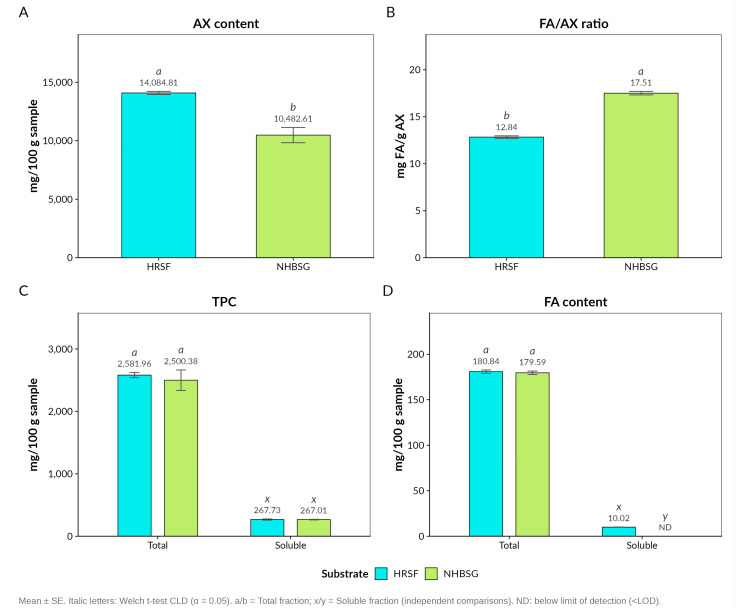
Arabinoxylan (AX), total phenolics (TPC), ferulic acid (FA), and feruloylation degree (FA/AX) in brewer’s spent grain solid samples. (**A**) AX. (**B**) TPC. (**C**) FA. (**D**) FA/AX. HRSF, hydrolyzed residual solid fraction; NHBSG, non-hydrolyzed brewer’s spent grain; ND, not detected. Values were expressed as mean ± standard deviation (*n* = 3). Different letters indicate significant differences according to one-way ANOVA followed by Tukey’s test (*p* < 0.05). For TPC and FA content, different sets of letters (a–b and x–y) denote significant differences within the total and soluble fractions, respectively.

Regarding the presence of AX and their relationship with phenolic compounds, especially FA, HRSF showed a significantly higher AX content than NHBSG fraction (14,084.81 ± 185.72 vs. 10,482.62 ± 1306.51 mg/100 g, respectively), corresponding to a 34.36% increase (Figure 1). This apparent increase in the relative AX content of HRSF after enzymatic treatment results from the preferential removal of other cell wall components rather than an increase in the absolute amount of AX. Enzymatic hydrolysis of the lignocellulosic matrix released soluble compounds into HLF, including proteins, lipids, and dietary fiber (Table 1). Although approximately 24% of the AX initially present in NHBSG was solubilized, the total dietary fiber loss reached nearly 59%, indicating that non-AX fiber components, particularly insoluble fiber, were removed to a greater extent. Consequently, the HRSF contained a lower proportion of other fiber components of total dietary fiber than NHBSG, but a higher proportion of AX within the remaining fiber fraction. Although HRSF exhibited a higher relative AX content than NHBSG, as mentioned above, both fractions exhibited similar FA contents (180.84 ± 3.28 vs. 179.59 ± 3.30 mg/100 g) (Table S2). This result implies a significant decrease in the degree of fiber feruloylation (FA/AX) in the hydrolyzed solid, from 17.51 ± 0.32 to 12.84 ± 0.23 mg FA/g AX, suggesting that enzymatic hydrolysis promoted the partial release of FA into the liquid fraction through the cleavage of ester bonds linking FA to AX. In cereals, the degree of AX feruloylation is associated with fiber structural rigidity and solubility; therefore, its decrease suggests structural modifications within the hemicellulose matrix, reducing phenolic crosslinking [41,54] and potentially affecting both digestibility and fermentability [55]. In this context, AX present in the residual solid fraction may become more accessible to fermentation by the gut microbiota [56,57].

Additionally, the total (matrix-associated) and soluble (non-covalently bound) contents of TPC and FA in HRSF were determined and compared with those in NHBSG. The HRSF contained a total of 2581.96 ± 70.63 mg/100 g of matrix-associated polyphenols, of which 267.73 ± 12.80 mg/100 g were present in the soluble fraction (10.37%). This indicates that only a limited proportion of phenolic compounds are weakly associated with the cell wall matrix after hydrolysis, whereas most remain structurally bound, likely through ester linkages to polysaccharides such as AX, thereby limiting their accessibility to the gut microbiota in the absence of prior chemical, enzymatic, or microbial degradation [58,59,60]. NHBSG exhibited a limited soluble TPC fraction, corresponding to 10.90% of total TPC. However, although enzymatic hydrolysis did not change the total FA content significantly, as was also observed for TPC, the soluble FA fraction showed an upward trend in HRSF compared with NHBSG. This finding suggests structural modifications in the hemicellulosic matrix induced by enzymatic hydrolysis, potentially improving microbial accessibility to feruloylated AX and colonic fermentability [23,55].

The accessibility of AX and phenolic compounds in lignocellulosic matrices is closely associated with their antioxidant capacity [61,62]. Therefore, the ORAC antioxidant activity of HRSF and NHBSG was compared, revealing that HRSF exhibited significantly higher antioxidant activity than NHBSG (64,825.35 ± 4011.24 vs. 45,352.96 ± 10,588.50 μmol TE/100 g, respectively; *p* = 0.041). The greater antioxidant capacity observed in HRSF may indicate increased structural accessibility in BSG after enzymatic hydrolysis. Enzymatic treatments applied to BSG have been associated with enhanced antioxidant activity due to the disruption of the plant cell wall and the release of bioactive compounds covalently bound to the matrix and/or physically trapped within its porous structure [63,64,65].

**Figure 2:**
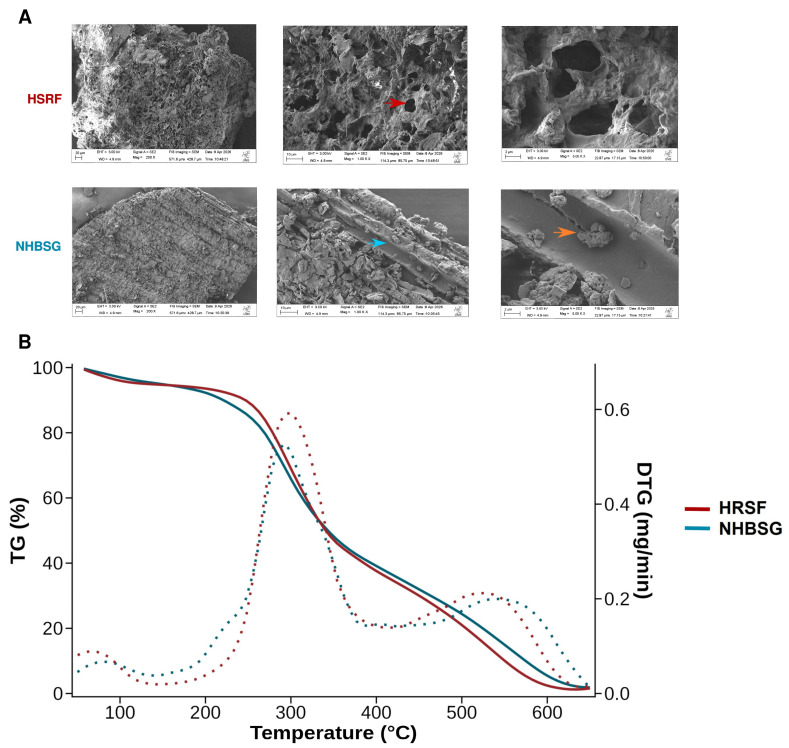
Structural modifications in brewer’s spent grain solid samples. (**A**) SEM micrographs of the hydrolyzed residual solid fraction (HRSF) and non-hydrolyzed brewer’s spent grain (NHBSG) at magnifications ranging from 200× to 5000×. Colored arrows indicate distinct surface features of BSG: red, pores; light blue, filamentous microfibrillar structures; yellow, irregular aggregates. (**B**) Thermogravimetric (TG, solid lines) and derivative thermogravimetric (DTG, dotted lines) curves of NHBSG and HRSF. DTG curves show the temperature-dependent mass loss rate, highlighting the main degradation stages of lignocellulosic components and residual carbonaceous material.

Structural modifications in HRSF compared to NHBSG were evaluated by SEM and TGA/DTG (Figure 2). NHBSG exhibited a relatively continuous and compact surface morphology, with filamentous microfibrillar structures and irregular granular aggregates, similar to those previously described by Outeiriño et al. (2019) [66]. In contrast, HRSF showed an eroded and irregular outer surface, increased porosity, cavity formation, partial detachment of structural layers, and a reduced abundance of granular aggregates (Figure 2A). Similar morphological alterations have been previously reported in cereal matrices subjected to enzymatic treatments, where degradation of the polymeric network leads to increased surface disruption and porosity [23,67,68]. These observations indicate greater disruption of the plant cell wall matrix following enzymatic hydrolysis, likely associated with the partial removal of hemicellulosic polysaccharides, thereby increasing exposure of the residual lignocellulosic fiber network and enhancing structural accessibility.

TG/DTG analysis revealed that the overall thermal stability of HRSF did not differ significantly from that of NHBSG (mean DTG, *p* = 0.97). However, differences were observed in the shape and distribution of thermal degradation profiles, with HRSF exhibiting faster thermal degradation and a higher maximum mass-loss rate (Figure 2B). Both samples displayed the typical lignocellulosic degradation pattern in DTG curves, including an initial dehydration stage followed by the main pyrolytic decomposition region between 200 and 500 °C [32,69,70]. Nevertheless, HRSF exhibited a more pronounced DTG peak in the hemicellulose-associated region around 280–320 °C, with higher intensity, a narrower profile, and a slight shift toward higher temperatures than NHBSG. These changes suggest enhanced thermal accessibility and possible structural reorganization of the remaining polysaccharide matrix in HRSF. In addition, within the lignin-associated region (>400 °C), HRSF exhibited a less pronounced degradation transition than NHBSG, which maintained a broader degradation profile with a more evident shoulder or secondary peak around 480–560 °C. These results suggest that NHBSG has a more preserved lignocellulosic structure, possibly due to stronger lignin–hemicellulose interactions. In contrast, HRSF presented a more disrupted polymeric network with lower resistance to thermal decomposition. Similar structural destabilization and increased exposure of the cellulosic fraction following partial hemicellulose removal have also been reported in chemically treated BSG matrices [23]. Overall, these findings are consistent with the SEM observations showing increased porosity and disruption of the plant cell wall structure in HRSF, likely associated with partial removal of hemicellulosic polysaccharides and subtle reorganization of the lignocellulosic matrix.

### 3.2. SCFA Production During In Vitro Colonic Fermentation

**Figure 3:**
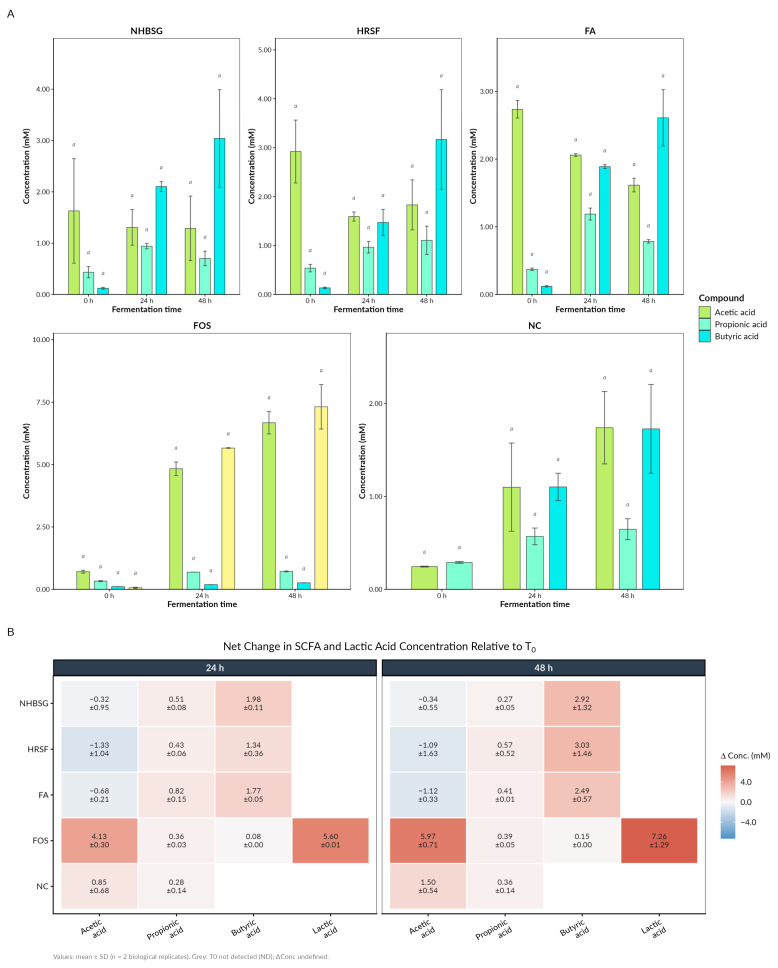
Individual short-chain fatty acid (SCFA) profiles after in vitro colonic fermentation of brewer’s spent grain solid samples. (**A**) Temporal dynamics of SCFA and lactic acid concentrations across fermentation substrates. (**B**) Heatmap showing the mean concentration change relative to T_0_ (Δ concentration = concentration_t − concentration_T_0_) for each substrate × compound combination at 24 h (left) and 48 h (right). Positive values (red) indicate net production, whereas negative values (blue) indicate net consumption relative to the initial concentration. HRSF, hydrolyzed residual solid fraction; NHBSG, non-hydrolyzed brewer’s spent grain; FA, ferulic acid; FOS, fructooligosaccharides; NC, negative control. Values are expressed as mean ± standard deviation (*n* = 2 biological replicates). Letters indicate significant differences among time points within each substrate according to one-way ANOVA followed by Dunn’s post hoc test with compact letter display (CLD) and Benjamini–Hochberg correction (*p* < 0.05). Gray cells indicate compounds not detected at T0, for which Δ concentration could not be calculated.

BSG solid samples (HRSF and NHBSG) were subjected to in vitro gastrointestinal digestion (INFOGEST) alongside a 0.01 g/L FA solution as pure FA control and then incubated in an in vitro colonic fermentation system with FOS and NC as positive and negative controls, respectively. SCFA concentrations and pH were measured at baseline, 24 h, and 48 h, revealing time-dependent fermentation profiles (Figure 3A).

HRSF fermentation showed decreased acetic acid (37.33% reduction to 1.83 ± 0.72 mM), slightly increased propionic acid (0.54 ± 0.11 to 1.11 ± 0.41 mM), and elevated butyric acid (22.6-fold increase to 3.17 ± 1.44 mM at 48 h). NHBSG maintained stable acetic acid levels (1.29 ± 0.89 mM), slightly increased propionic acid (0.43 ± 0.15 to 0.70 ± 0.20 mM), and elevated butyric acid (25.3-fold increase to 3.04 ± 1.35 mM), consistent with previous BSG fermentation studies reporting SCFA concentrations ranging from 3 to 20 mM [13,25,71]. Pure FA control demonstrated decreased acetic acid (40.9% to 1.62 ± 0.14 mM), increased propionic acid (0.37 ± 0.03 to 0.79 ± 0.04 mM), and elevated butyric acid (21.8-fold increase to 2.61 ± 0.59 mM). FOS fermentation showed acetic acid increases (9.4-fold to 6.68 ± 0.63 mM) with propionic and butyric acids remaining below 1 mM, while uniquely producing lactic acid (122-fold increase to 7.32 ± 1.26 mM). NC showed only moderate increases across all SCFAs, reaching maximum concentrations of ~1.7 mM.

Distinct SCFA distributions were observed after 48 h of fermentation depending on the substrate. HRSF, NHBSG, and the pure FA control exhibited fermentation profiles characterized by substantial butyrate accumulation, whereas FOS fermentation was characterized primarily by acetate and lactate accumulation. Collectively, these observations suggest that the feruloylated substrates were associated with a butyrate-oriented fermentation pattern under the conditions evaluated.

The substrate-dependent fermentation patterns are further illustrated in Figure 3B, which presents the net production (positive values) or consumption (negative values) of each organic acid as the change in concentration relative to baseline (Δ conc relative to T_0_) throughout fermentation. The acetate- and lactate-oriented profile observed for FOS was consistent with the metabolite distributions shown in Figure 3A. In contrast, the feruloylated substrates (HRSF, NHBSG, and the pure FA control) exhibited progressive net butyrate accumulation throughout fermentation, particularly after 48 h of incubation. Among these substrates, HRSF displayed the largest net increase in butyric acid concentration at the end of the fermentation period.

The butyric acid accumulation observed in the present study is consistent with previous reports identifying cereals as substrates that promote butyrogenic fermentation by the gut microbiota [72]. Recent in vitro studies have demonstrated enhanced butyrate production during the fermentation of barley [73,74], wheat–quinoa mixtures [75], and oat-based substrates [76], among others. Similarly, cereal-derived AX from rice [55], maize [55], wheat bran [56], and wheat-derived AX [77] have also been shown to enhance butyrate production during in vitro colonic fermentation. Furthermore, studies on feruloylated arabinoxylan-derived oligosaccharides (AXOSs) have reported enhanced butyric acid production during colonic fermentation [78,79], suggesting that both polysaccharide structure and degree of feruloylation may influence microbial fermentation patterns. In the present study, HRSF displayed the largest net increase in butyric acid concentration and exhibited a lower degree of feruloylation than NHBSG (Figure 1B). Although the mechanisms underlying this observation remain to be elucidated, a lower degree of feruloylation could increase the accessibility of the AX backbone to xylanolytic enzymes produced by the gut microbiota, thereby facilitating substrate degradation and subsequent butyrate formation [78]. Collectively, these findings support previous evidence that cereal-processing by-products promote butyrogenic fermentation in vitro and further suggest that structural features of AX, particularly the degree of feruloylation, may be an important determinant of their butyrogenic potential.

### 3.3. Modulation of the Human Gut Microbiota Composition During In Vitro Colonic Fermentation

**Figure 4:**
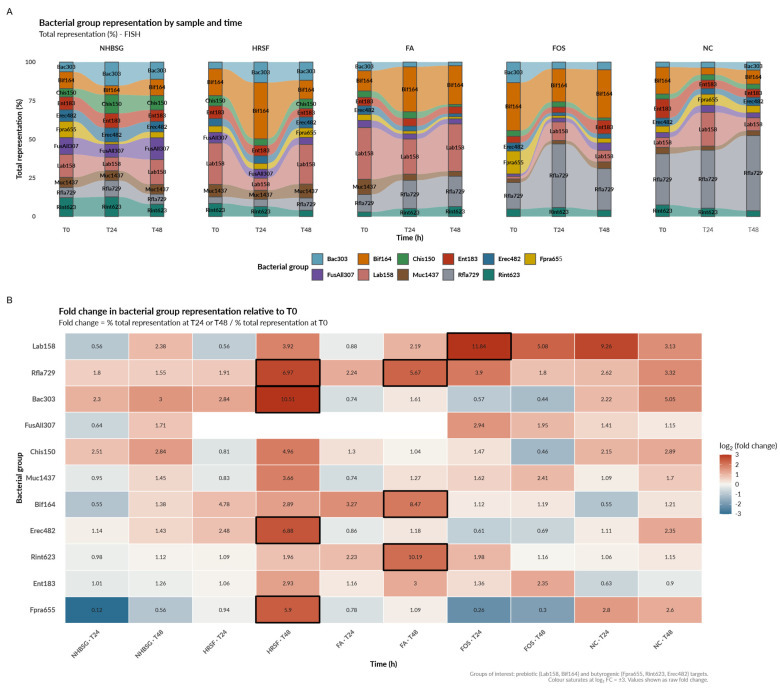
Relative abundance modulation of targeted human gut bacterial groups during in vitro colonic fermentation of brewer’s spent grain solid samples. (**A**) Relative abundance (RA) profile of the targeted bacterial groups determined by fluorescence in situ hybridization coupled to flow cytometry (FISH-FC) at 0, 24, and 48 h of fermentation, expressed as compositional percentage. (**B**) Heatmap of the fold change (FC) in the RA of the different bacterial groups at 24 and 48 h relative to time 0, expressed as log2(FC). HRSF, hydrolyzed residual solid fraction; NHBSG, non-hydrolyzed brewer’s spent grain; FA, ferulic acid; FOS, fructooligosaccharides; NC, negative control. Bacterial groups: Lab158 (*Lactobacillus*/*Enterococcus*), Bif164 (*Bifidobacterium*), Rfla729 (*R. albus*/*R. flavefaciens*), Muc1437 (*A. muciniphila*), Erec482 (*E. rectale*/*C. coccoides*), Rint623 (*R. intestinalis*/*R. cecicola*), Fpra655 (*F. prausnitzii*), Bac303 (*Bacteroides*/*Prevotella*), Chis150 (*C. histolyticum*), Ent183 (*Enterobacteriaceae*), and FusAll307 (*Fusobacterium*). Values are expressed as mean (*n* = 2).

The composition of targeted gut bacterial groups during fermentation of BSG solid substrates showed temporal modulation at 24 and 48 h (Figure 4A). Overall, these samples exhibited dynamic shifts in the relative composition of the eleven bacterial groups evaluated over time. However, the changes observed in HRSF were more pronounced, showing higher relative abundances of bacterial groups commonly regarded as relevant biomarkers of prebiotic activity, such as *Lactobacillus* and *Bifidobacterium*, which were also prominently represented during FA fermentation. Notably, potentially undesirable groups, including the *C. histolyticum* group, *Enterobacteriaceae*, and *Fusobacterium*, were underrepresented in HRSF and FA fermentations. In addition, a greater relative abundance of *A. muciniphila* and *Bacteroides* was observed in HRSF and NHBSG compared to the other substrates (Figure 4A).

In previously reported in vitro colonic fermentation systems, BSG has shown high fermentability, primarily due to its high dietary fiber content, particularly AX [71]. Several studies have demonstrated that the AX fermentation by the gut microbiota promotes a positive modulation of the microbial community, enhancing the growth of beneficial bacteria such as *Lactobacillus* and *Bifidobacterium* [12,25,71,80,81], which are primarily involved in the saccharolytic fermentation of non-digestible carbohydrates. The increase in *Bifidobacterium* has been consistently reported during in vitro colonic fermentation of AX and AXOS obtained from this agro-industrial residue [12,13,14,81,82]. This genus can cleave the structures of both AX [83] and AXOS [84]. In addition, AX extracted from BSG and other cereals has been shown to promote the growth of genera such as *Bacteroides* and *Prevotella* [13,82,85,86,87,88], which exhibit well-established xylanolytic capabilities and play a key role in the primary degradation of complex polysaccharides [89]. Regarding *A. muciniphila*, it is recognized as a beneficial gut bacterium due to its role in mucin degradation, preservation of intestinal barrier integrity, and modulation of host immune responses. Although high-fiber diets have been associated with increased *Akkermansia* abundance [90,91,92], its response is not typically dominant in lignocellulosic substrates. Nevertheless, its increase has been reported in BSG fermentations [81], and in fermentations of AX derived from this by-product [12] and feruloylated AX [93,94], suggesting that its enrichment may reflect indirect ecological shifts as a product of bacterial community modulation.

To better characterize substrate-dependent microbial modulation, temporal changes in bacterial relative abundance were further expressed as log2 fold change relative to baseline. This analysis enabled a more detailed assessment of bacterial groups selectively enriched or reduced during fermentation (Figure 4B). Overall, HRSF showed the strongest microbiota-modulating effect among the evaluated substrates. At 48 h, the HRSF induced marked increases in groups known for their butyrogenic capacity, such as *F. prausnitzii* (+5.90) and *E. rectale* (+6.88), suggesting fermentation directed towards butyrate production. *F. prausnitzii* is recognized as one of the main butyrate producers in the human colon [95], and together with *Eubacterium*, its increase has been consistently reported in fermentations of AX-rich prebiotic fibers derived from cereal by-products. For example, in vitro fermentation of BSG induced a delayed increase in *Eubacterium* and *Faecalibacterium*, which was strongly correlated with butyrate production [81]. Similarly, fermentation of wheat bran-derived AX selectively promoted the growth of *Faecalibacterium* [56]. In agreement with these findings, in vivo studies have also reported an increase in *E. rectale* following the consumption of AX-rich diets [96]. More broadly, systematic reviews of human intervention studies have shown that the consumption of whole grains and cereal-derived fibers—particularly wheat bran, barley, and rye, and to a lesser extent oats, brown rice (or rice bran), and resistant starch-rich maize—is associated with the enrichment of butyrate-producing bacterial groups, especially *Faecalibacterium*, *Eubacterium*, and *Roseburia* [97,98].

The same sample, HRSF, also showed increases in *Bacteroides* (+10.51) and *R. albus/R. flavefaciens* (+6.97) at 48 h. *Bacteroides* is a bacterial group known to be involved in the degradation of feruloylated AX [99]. On the other hand, the *R. albus*/*R. flavefaciens* group is associated with the degradation of structural plant polysaccharides, particularly cellulose and hemicelluloses, and is considered an important fibrolytic bacterial group during dietary fiber fermentation [100].

Notably, the *R. albus*/*R. flavefaciens* group also showed a marked increase during FA fermentation (+5.67 at 48 h), together with increases in *Bifidobacterium* (+8.47) and *R. intestinalis*/*R. cecicola* (+10.19). The genus *Roseburia* significantly contributes to colonic butyrate pools through the fermentation of dietary fibers and cross-feeding interactions [101] and can degrade AX via the activity of xylanases, α-L-arabinofuranosidases, and β-xylosidases [102]. The selective enrichment of *Ruminococcus* and butyrogenic bacterial groups during HRSF and FA fermentation suggests that these taxa may be associated with fermentation processes occurring in the presence of this hydroxycinnamic acid. In this context, although the prebiotic activity of BSG has mainly been attributed to its AX and feruloylated AX content, emerging evidence suggests that free FA may also increase the relative abundance of beneficial bacterial groups, including *Bacteroides* spp. and *Lactobacillus* spp. [46,53,103,104].

On the other hand, contrary to HRSF, NHBSG showed more moderate changes without consistently stimulating butyrogenic bacterial groups. As expected, FOS markedly increased the abundance of *Lactobacillus* (+11.84), while NC showed only minor variations associated with the basal dynamics of the microbiota.

Overall, FISH-FC results indicate that the HRSF retained structural characteristics that may enhance microbial accessibility to fermentable polysaccharides, thereby favoring broader and more functionally relevant microbial modulation compared to NHBSG. The microbial modulation observed in the residual solid correlated closely with the previously described SCFA profile, particularly with the increase in butyrate production. The increase in *F. prausnitzii* and *E. rectale*, both recognized butyrate-producing bacteria, provides an ecological basis for explaining the increased levels of this metabolite. In addition, potential microbiota modulation in HRSF involved the enrichment of fibrolytic bacterial groups, including *Bacteroides* and *Ruminococcus*, which are associated with the degradation of complex plant polysaccharides. These findings suggest that the structural matrix of the residual solid, rich in partially feruloylated complex polysaccharides, favors fermentative pathways associated with specialized butyrogenic and fibrolytic bacteria. It is important to note that the feruloylation of cereal polysaccharides is a key determinant of fermentability, as it modulates microbial enzymatic accessibility and fermentation kinetics in a structure- and source-dependent manner. Previous studies on cereal polysaccharides have shown that the impact of the degree of feruloylation on microbial accessibility and fermentability is context-dependent. For instance, deferuloylation of maize AX has been reported to increase butyrate production compared with native maize AX, an effect associated with shifts in microbial composition, including changes in the abundance of *B. ovatus* and *Blautia*. In contrast, deferuloylation of rice AX resulted in lower butyrate production than native rice AX, accompanied by a reduction in *F. prausnitzii* [55]. Furthermore, the degree of feruloylation in corn bran-derived AX has been shown to directly influence fermentation properties, including kinetics [105]. The presence of specific esterases in human gut microbial groups capable of degrading AX, such as *Bacteroides*, suggests that FA desesterification can facilitate enzymatic access to complex polysaccharide structures during degradation [106]. In this context, FA cross-linking has been shown to reduce the rate of FA fermentation [105], likely due to structural constraints that limit enzyme accessibility.

Collectively, these findings reinforce the prebiotic potential of HRSF, particularly its ability to modulate the gut microbiota toward a more beneficial and butyrogenic microbial profile. This is relevant when considering HRSF as a potential flour with prebiotic properties that differ from those most reported for BSG, which are predominantly bifidogenic and propionogenic, characterized by increased acetate and propionate production with only limited butyrate formation [107,108]. Among the available in vitro fermentation studies on BSG, only a limited number have reported a clear butyrogenic response [81]. Although the butyrogenic effect observed in this study was moderate, it is biologically relevant given the central role of butyrate in maintaining intestinal barrier integrity, modulating immune responses, and supporting colonic health [109]. Emerging evidence also links butyrate to host metabolic regulation and the gut–brain axis [110]. These results therefore support the need for in vivo studies to evaluate whether such effects can be translated into measurable health outcomes in humans. In addition to its prebiotic potential, BSG exhibits relevant techno-functional properties, including high water-holding and fat-binding capacities [108]. Accordingly, BSG-derived ingredients have been incorporated into a wide range of food products, such as bread, cookies, baked snacks, pasta, noodles, muffins and yogurt, among others [111]. In many applications, BSG enables partial replacement of conventional ingredients while contributing to reductions in fat and sugar content, supporting the development of healthier formulations [111,112]. From a biorefinery perspective, this study demonstrates, to the best of our knowledge, for the first time that the solid fraction remaining after enzymatic hydrolysis of BSG shows a tendency to promote butyrogenic activity. This highlights a previously unexploited opportunity for the valorization of this agro-industrial by-product and expands its potential use as a functional food ingredient.

## 4. Conclusions

The residual solid obtained after standardized enzymatic treatment of BSG shows promising prebiotic properties, characterized by a high content of fermentable dietary fiber and hydroxycinnamic acids, particularly ferulic acid, as well as structural modifications that improve the accessibility of the polysaccharide matrix relative to NHBSG.

In vitro colonic fermentation of HRSF demonstrated the potential to modulate the gut microbiota, leading to increased production of SCFAs, especially butyrate, which is crucial for maintaining intestinal health. This effect was accompanied by an increased abundance of beneficial butyrate-producing bacteria, including *F. prausnitzii* and *E. rectale*. Additionally, there was a notable rise in other beneficial bacterial groups, such as *Bacteroides*, *Lactobacillus*, *and Bifidobacterium* spp., which are known for their roles in promoting gut health and enhancing the fermentation of dietary fibers.

Overall, these findings support the comprehensive valorization of brewer’s spent grain following biorefinery processes. In addition to generating a liquid fraction rich in bioactive compounds, the residual solid fraction could be further exploited as a functional ingredient with microbiota-modulating potential, contributing to a circular-economy strategy in food technology.

From an application perspective, the enzymatically hydrolyzed residual solid is a promising fiber-rich ingredient candidate, valued not only for total dietary fiber content but also for the accessibility and fermentability of specific polysaccharide fractions. Its arabinoxylan enrichment and improved microbial fermentation outcomes support potential incorporation into gut health-oriented food matrices, including bakery, cereal, and nutritional products. Controlled enzymatic processing appears to enhance the physiological functionality of agro-industrial by-products by modulating fiber structure and promoting selective microbial responses.

Finally, these findings support the physiological relevance of HRSF fermentation behavior and its potential as a prebiotic substrate. Overall, they provide a basis for further in vitro and in vivo studies and highlight their potential as a functional food ingredient. Future research should address technological performance, sensory acceptance, dose–response relationships, in vivo validation, and interindividual microbiota variability to guide personalized nutrition applications.

## Figures and Tables

**Table 1:** Chemical composition of the hydrolyzed fractions of brewer’s spent grain.

Parameter	HRSF [g/100 g]	HLF [g/100 g]	NHBSG [g/100 g]
Moisture	3.91 ± 0.04	97.90 ± 0.01	3.09 ± 0.05
Ash	2.90 ± 0.04	0.07 ± 0.01	2.73 ± 0.02
Ether extract	6.18 ± 0.00	0.20 ± 0.01	5.98 ± 0.04
Protein	18.37 ± 0.14	0.14 ± 0.01	19.85 ± 0.23
Total dietary fiber	43.37	0.71	48.67
Insoluble fiber	41.71 ± <0.01	0.29 ± 0.00	46.08 ± 0.00
Soluble fiber	1.66 ± 0.00	0.42 ± 0.00	2.59 ± 0.00
Arabinoxylans	14.08 ± 0.19	2.60 ± 0.19	10.48 ± 1.31
Available carbohydrates	25.27	0.98	19.68
Total sugars	11.98 ± 0.13	0.50 ± 0.08	7.91 ± 0.10

HRSF, hydrolyzed residual solid fraction; HLF, hydrolyzed liquid fraction; NHBSG, non-hydrolyzed brewer’s spent grain. Values were expressed as mean ± standard deviation (*n* = 3). Available carbohydrates were calculated by difference. Total dietary fiber was calculated as the sum of insoluble and soluble dietary fiber.

## Data Availability

The original contributions presented in the study are included in the article/Supplementary Materials; further inquiries can be directed to the corresponding authors.

## Supplementary Materials

The following supporting information can be downloaded at https://www.mdpi.com/article/10.3390/foods15132378/s1. Table S1: Validation parameters of the HPLC method for the determination of hydroxycinnamic acids. Table S2: Phenolic profile of the hydrolyzed fractions of brewer’s spent grain. Table S3: Net total short-chain fatty acid (SCFA) accumulation (mM) over time during in vitro colonic fermentation of brewer’s spent grain solid samples. Table S4: Chemical composition of different batches of brewer’s spent grain from the same IPA beer type used in this study. Table S5: Percentage distribution of arabinoxylan content in the enzymatically hydrolyzed fractions of brewer’s spent grain from two batches of the same IPA beer type used in this study.

## References

[1] Nyhan L., Sahin A.W., Schmitz H.H., Siegel J.B., Arendt E.K. (2023). Brewers’ spent grain: An unprecedented opportunity to develop sustainable plant-based nutrition ingredients addressing global malnutrition challenges. J. Agric. Food Chem..

[2] Ikram S., Huang L., Zhang H., Wang J., Yin M. (2017). Composition and nutrient value proposition of brewers’ spent grain. J. Food Sci..

[3] Henkin J.M., Mainali K., Sharma B.K., Yadav M.P., Ngo H., Sarker M.I. (2025). A review of chemical and physical analysis, processing, and repurposing of brewers’ spent grain. Biomass.

[4] Bonifácio-Lopes T., Vilas-Boas A.A., Coscueta E.R., Costa E.M., Silva S., Campos D., Teixeira J.A., Pintado M. (2020). Bioactive extracts from brewer’s spent grain. Food Funct..

[5] Chetrariu A., Dabija A. (2020). Brewer’s spent grains: Possibilities of valorization, a review. Appl. Sci..

[6] Tapia D., Quiñones J., Martínez A., Millahual E., Campagnol P.C.B., Sepúlveda N., Diaz R. (2025). The silent revolution of brewer’s spent grain: Meat/food innovations through circularity, resource recovery, and nutritional synergy-a review. Foods.

[7] Soto-Maldonado C., Espinosa A., Bucarey J.L., Fuentes L. (2026). Functional benefits of brewer’s spent grain and the challenge of developing food ingredients for human health. Antioxidants.

[8] Bartolomé B., Santos M., Jiménez J.J., del Nozal M.J., Gómez-Cordovés C. (2002). Pentoses and hydroxycinnamic acids in brewer’s spent grain. J. Cereal Sci..

[9] Mandalari G., Faulds C.B., Sancho A.I., Saija A., Bisignano G., LoCurto R., Waldron K.W. (2005). Fractionation and characterization of arabinoxylans from brewers’ spent grain and wheat bran. J. Cereal Sci..

[10] Nkurunziza D., Kumorkiewicz-Jamro A., Yu L., Duggan J., Collins H., Pukala T., Bulone V., Coad B.R. (2025). Differential release of biologically active cell-wall-bound phenolics from brewer’s spent grain using conventional and deep eutectic solvents. ACS Sustain. Chem. Eng..

[11] Zheng M., Liu Y., Zhang G., Yang Z., Xu W., Chen Q. (2024). The antioxidant properties, metabolism, application and mechanism of ferulic acid in medicine, food, cosmetics, livestock and poultry. Antioxidants.

[12] Lynch K.M., Strain C.R., Johnson C., Patangia D., Stanton C., Koc F., Gil-Martinez J., O’Riordan P., Sahin A.W., Ross R.P. (2021). Extraction and characterisation of arabinoxylan from brewers’ spent grain and investigation of microbiome modulation potential. Eur. J. Nutr..

[13] Reis S.F., Gullón B., Gullón P., Ferreira S., Maia C.J., Alonso J.L., Domingues F.C., Abu Ghannam N. (2014). Evaluation of the prebiotic potential of arabinoxylans from brewer’s spent grain. Appl. Microbiol. Biotechnol..

[14] Sajib M., Falck P., Sardari R.R.R., Mathew S., Grey C., Nordberg Karlsson E., Adlercreutz P. (2018). Valorization of brewer’s spent grain to prebiotic oligosaccharide: Production, xylanase catalyzed hydrolysis, in-vitro evaluation with probiotic strains and in a batch human fecal fermentation model. J. Biotechnol..

[15] Bonifácio-Lopes T., Teixeira J.A., Pintado M. (2020). Current extraction techniques towards bioactive compounds from brewer’s spent grain—A review. Crit. Rev. Food Sci. Nutr..

[16] Bonifácio-Lopes T., Castro L.M.G., Vilas-Boas A., Campos D., Teixeira J.A., Pintado M. (2023). Impact of gastrointestinal digestion simulation on brewer’s spent grain green extracts and their prebiotic activity. Food Res. Int..

[17] Al-Shwafy K.W.A., Chadni M., Abg Zamari M.H.H., Ioannou I. (2023). Enzymatic extraction of ferulic acid from brewer’s spent grain: Effect of physical pretreatments and optimization using design of experiments. Biocatal. Agric. Biotechnol..

[18] Bucci P.L., Martins P., Muñoz R., Meyer A.S. (2024). Synergistic enzyme-assisted release of ferulic acid from brewer’s spent grain. Process Biochem..

[19] Naibaho J., Korzeniowska M., Phimolsiripol Y., Gavahian M. (2025). Value-added compounds from brewers’ spent grain to develop bioactive-enriched food ingredients: Recent advances in sustainable extraction and considerations for industrial application. Food Biosci..

[20] Sibhatu H.K., Jabasingh S.A., Yimam A., Ahmed S. (2021). Ferulic acid production from brewery spent grains, an agro-industrial waste. LWT.

[21] Bucci P.L., Casas A., Martins P., Meyer A.H., Cantero D.A., Muñoz R. (2024). A comparative assessment of treatment methods to release ferulic and p-coumaric acids from brewer’s spent grains. Waste Manag..

[22] Shen Y., Abeynayake R., Sun X., Ran T., Li J., Chen L., Yang W. (2019). Feed nutritional value of brewers’ spent grain residue resulting from protease aided protein removal. J. Anim. Sci. Biotechnol..

[23] Wagner E., Pería M.E., Ortiz G.E., Rojas N.L., Ghiringhelli P.D. (2021). Valorization of brewer’s spent grain by different strategies of structural destabilization and enzymatic saccharification. Ind. Crops Prod..

[24] Zeng J., Huang W., Tian X., Hu X., Wu Z. (2021). Brewer’s spent grain fermentation improves its soluble sugar and protein as well as enzymatic activities using *Bacillus velezensis*. Process Biochem..

[25] Bonifácio-Lopes T., Catarino M.D., Vilas-Boas A.A., Ribeiro T.B., Campos D.A., Teixeira J.A., Pintado M. (2022). Impact of circular brewer’s spent grain flour after in vitro gastrointestinal digestion on human gut microbiota. Foods.

[26] Báez J., Fernández-Fernández A.M., Briozzo F., Díaz S., Dorgans A., Tajam V., Medrano A. (2021). Effect of enzymatic hydrolysis of brewer’s spent grain on bioactivity, techno-functional properties, and nutritional value when added to a bread formulation. Biol. Life Sci. Forum.

[27] Paiva C.F., Almeida T.S.F., Arelhano G.E., Alvarado A.V.R., Menezes M.B., Argandoña E.J.S., Gomes I.L.A., Moya A.M.T.M., Filho P.S.L., Santos E.F.D. (2024). Potential of brewer’s spent grain as a nutritional ingredient in bakery products. Plant Foods Hum. Nutr..

[28] Liu K., Xie Z., Huang Y., Suo H., Zhao G., Wang D. (2026). Brewers’ spent grain reborn via cellulase–xylanase hydrolysis and *Lactobacillus acidophilus* fermentation and their application in bread. Innov. Food Sci. Emerg. Technol..

[29] López-Linares J.C., Lucas S., García-Cubero M.T., Jiménez J.J., Coca M. (2020). A biorefinery based on brewer`s spent grains: Arabinoxylans recovery by microwave assisted pretreatment integrated with butanol production. Ind. Crops Prod..

[30] Faulds C.B., Sancho A.I., Bartolomé B. (2002). Mono- and dimeric ferulic acid release from brewer’s spent grain by fungal feruloyl esterases. Appl. Microbiol. Biotechnol..

[31] Faulds C.B., Mandalari G., LoCurto R., Bisignano G., Waldron K.W. (2004). Arabinoxylan and mono- and dimeric ferulic acid release from brewer’s grain and wheat bran by feruloyl esterases and glycosyl hydrolases from *Humicola insolens*. Appl. Microbiol. Biotechnol..

[32] Marchese L., Kühl K.I.P., da Silva J.C.G., Mumbach G.D., Alves R.F., Alves J.L.F., Di Domenico M. (2024). Exploring bioenergy prospects from malt bagasse: Insights through pyrolysis with multi-component kinetic analysis and thermodynamic parameter estimation. Renew. Energy.

[33] Van de Velden M., Baeyens J., Brems A., Janssens B., Dewil R. (2010). Fundamentals, kinetics and endothermicity of the biomass pyrolysis reaction. Renew. Energy.

[34] El-Sayed S.A., Khass T.M., Mostafa M.E. (2024). Thermal degradation behaviour and chemical kinetic characteristics of biomass pyrolysis using TG/DTG/DTA techniques. Biomass Convers. Biorefinery.

[35] Horwitz W., Latimer G.W. (2005). Official Methods of Analysis of AOAC International.

[36] Kareparamban J.A., Nikam P.H., Jadhav A.P., Kadam V.J. (2013). A Validated High-performance liquid chromatography method for estimation of ferulic acid in Asafoetida and polyherbal preparation. Indian J. Pharm. Sci..

[37] Nadal J.M., Toledo M.D.G., Pupo Y.M., de Paula J.P., Farago P.V., Zanin S.M. (2015). A stability-indicating HPLC-DAD method for determination of ferulic acid into microparticles: Development, validation, forced degradation, and encapsulation efficiency. J. Anal. Methods Chem..

[38] Prior R.L., Wu X., Schaich K. (2005). Standardized methods for the determination of antioxidant capacity and phenolics in foods and dietary supplements. J. Agric. Food Chem..

[39] Valdenegro M., Bernales M., Knox M., Vinet R., Caballero E., Ayala-Raso A., Kučerová D., Kumar R., Viktorová J., Ruml T. (2021). Characterization of fruit development, antioxidant capacity, and potential vasoprotective action of peumo (*Cryptocarya alba*), a native fruit of Chile. Antioxidants.

[40] Rose D.J., Inglett G.E. (2011). A method for the determination of soluble arabinoxylan released from insoluble substrates by xylanases. Food Anal. Methods.

[41] Carvajal-Millan E., Landillon V., Morel M.-H., Rouau X., Doublier J.-L., Micard V. (2005). Arabinoxylan gels: Impact of feruloylation degree on structure and properties. Biomacromolecules.

[42] Singleton V.L., Orthofer R., Lamuela-Raventós R.M. (1999). Analysis of total phenols and other oxidation substrates and antioxidants by means of folin-ciocalteu reagent. Methods Enzymol..

[43] Minekus M., Alminger M., Alvito P., Ballance S., Bohn T., Bourlieu C., Carrière F., Boutrou R., Corredig M., Dupont D. (2014). A standardized static in vitro digestion method suitable for food—An international consensus. Food Funct..

[44] Silva G.S., Moreira F.I.N., de Albuquerque T.M.R., Abreu T.L., de Souza E.G.T., da Silva L.R., Marques A.D.J.d.F., Galvão M.d.S., Lima M.d.S., de Souza E.L. (2025). Microencapsulated phenolic compounds from organic coffee husk: Impacts on human gut microbiota and in vitro prebiotic potential. Food Res. Int..

[45] Albuquerque T.M.R., Magnani M., Lima M.d.S., Castellano L.R.C., Souza E.L. (2021). Effects of digested flours from four different sweet potato (*Ipomoea batatas* L.) root varieties on the composition and metabolic activity of human colonic microbiota in vitro. J. Food Sci..

[46] Lu S., Cheng D., Yao H., Wen Y., Yu Y., Li H., Wang J., Sun B. (2024). Cascade microbial metabolism of ferulic acid in vitro fermented by the human fecal inoculum. J. Agric. Food Chem..

[47] Aguirre M., Ramiro-Garcia J., Koenen M.E., Venema K. (2014). To pool or not to pool? Impact of the use of individual and pooled fecal samples for in vitro fermentation studies. J. Microbiol. Methods.

[48] Stewart M.L., Timm D.A., Slavin J.L. (2008). Fructooligosaccharides exhibit more rapid fermentation than long-chain inulin in an in vitro fermentation system. Nutr. Res..

[49] Sampaio K.B., Nascimento D.d.S., Garcia E.F., de Souza E.L. (2022). An outlook on fluorescent in situ hybridization coupled to flow cytometry as a versatile technique to evaluate the effects of foods and dietary interventions on gut microbiota. Arch. Microbiol..

[50] R Core Team (2026). R: A Language and Environment for Statistical Computing.

[51] Mussatto S.I., Dragone G., Roberto I.C. (2007). Ferulic and *p*-coumaric acids extraction by alkaline hydrolysis of brewer’s spent grain. Ind. Crops Prod..

[52] Ideia P., Sousa Ferreira I., Castilho P.C. (2020). A novel and simpler alkaline hydrolysis methodology for extraction of ferulic acid from brewer’s spent grain. Foods.

[53] Huang J., Sun Z., Zhang G., Zhang Z., Sun F., Han D., Wang J., Zhao J. (2025). Ferulic acid mediates microbial fermentation of arabinoxylan to enhance host immunity by suppressing TLR4/NF-κB signaling. Int. J. Biol. Macromol..

[54] Freeman J., Ward J.L., Kosik O., Lovegrove A., Wilkinson M.D., Shewry P.R., Mitchell R.A.C. (2017). Feruloylation and structure of arabinoxylan in wheat endosperm cell walls from RNAi lines with suppression of genes responsible for backbone synthesis and decoration. Plant Biotechnol. J..

[55] Li Z., Zhang H., He L., Hou Y., Che Y., Liu T., Xiong S., Zhang X., Luo S., Liu C. (2023). Influence of structural features and feruloylation on fermentability and ability to modulate gut microbiota of arabinoxylan in in vitro fermentation. Front. Microbiol..

[56] Demuth T., Edwards V., Bircher L., Lacroix C., Nyström L., Geirnaert A. (2021). In vitro colon fermentation of soluble arabinoxylan is modified through milling and extrusion. Front. Nutr..

[57] Zhang D., Rudjito R.C., Pietiäinen S., Chang S.-C., Idström A., Evenäs L., Vilaplana F., Jiménez-Quero A. (2023). Arabinoxylan supplemented bread: From extraction of fibers to effect of baking, digestion, and fermentation. Food Chem..

[58] Acosta-Estrada B.A., Gutiérrez-Uribe J.A., Serna-Saldívar S.O. (2014). Bound phenolics in foods, a review. Food Chem..

[59] Wang T., He F., Chen G. (2014). Improving bioaccessibility and bioavailability of phenolic compounds in cereal grains through processing technologies: A concise review. J. Funct. Foods.

[60] Nignpense B.E., Francis N., Blanchard C., Santhakumar A.B. (2021). Bioaccessibility and bioactivity of cereal polyphenols: A review. Foods.

[61] Bijalwan V., Ali U., Kesarwani A.K., Yadav K., Mazumder K. (2016). Hydroxycinnamic acid bound arabinoxylans from millet brans—Structural features and antioxidant activity. Int. J. Biol. Macromol..

[62] Wang L., Wang J., Wang J., Guo Z., Li Z., Qiu J., Wang L. (2024). Soluble and insoluble dietary fiber at different ratios: Hydration characteristics, rheological properties, and ameliorative effects on constipation. Food Chem. X.

[63] Verni M., Pontonio E., Krona A., Jacob S., Pinto D., Rinaldi F., Verardo V., Díaz-de-Cerio E., Coda R., Rizzello C.G. (2020). Bioprocessing of brewers’ spent grain enhances its antioxidant activity: Characterization of phenolic compounds and bioactive peptides. Front. Microbiol..

[64] Naibaho J., Korzeniowska M., Wojdyło A., Ayunda H.M., Foste M., Yang B. (2022). Techno-functional properties of protein from protease-treated brewers’ spent grain (BSG) and investigation of antioxidant activity of extracted proteins and BSG residues. J. Cereal Sci..

[65] Naibaho J., Wojdyło A., Korzeniowska M., Laaksonen O., Föste M., Kütt M., Yang B. (2022). Antioxidant activities and polyphenolic identification by UPLC-MS/MS of autoclaved brewers’ spent grain. LWT.

[66] Outeiriño D., Costa-Trigo I., Paz A., Deive F.J., Rodríguez A., Domínguez J.M. (2019). Biorefining brewery spent grain polysaccharides through biotuning of ionic liquids. Carbohydr. Polym..

[67] Douge M., Nonus M., Thomasset T., Teissier P., Barbeau J.-Y. (2004). ESEM study of the effects of hydrolytic enzymes on wheat bran structure. Microsc. Anal..

[68] Pasha I., Ahmad F., Usman M. (2021). Elucidation of morphological characteristics, crystallinity, and molecular structures of native and enzyme modified cereal brans. J. Food Biochem..

[69] Borel L.D.M.S., Lira T., Ribeiro J.A., Ataíde C.H., Barrozo M.A.S. (2018). Pyrolysis of brewer’s spent grain: Kinetic study and products identification. Ind. Crops Prod..

[70] Gomez-Hernandez E., Hernández-Hernández E., Castro-Rosas J., Vázquez-García R.A., Cadena-Ramírez A., Jiménez-Villeda B.E., Gomez-Aldapa C.A. (2025). High-energy milling as a pre-treatment alternative for lignocellulosic fibers derived from brewer’s spent grain. Polymers.

[71] Calvete-Torre I., Sabater C., Montilla A., Moreno F.J., Riestra S., Margolles A., Ruiz L. (2023). Physicochemical characterization and microbiota modulatory potential of brewer’s spent grain and arabinoxylan-derived fractions: A valorization study. LWT.

[72] Knudsen K.E.B. (2015). Microbial degradation of whole-grain complex carbohydrates and impact on short-chain fatty acids and health. Adv. Nutr..

[73] Nagata R., Morioka M., Fukuma N., Hayashi K., Iwami A., Han K.-H., Fukushima M. (2022). In vitro colonic fermentation characteristics of barley-koji differ from those of barley. Biosci. Biotechnol. Biochem..

[74] Martínez-Subirá M., Cortijo Alfonso M.E., Friero I., Macià A., Pena R.N., Molinero N., Moreno-Arribas M.V., Rubió-Piqué L., Moralejo M. (2026). Barley extrudates modulate the gut microbiome–metabolome axis in vitro through β-glucan fermentation and polyphenol biotransformation. Food Funct..

[75] Xu L., Yu Q., Ma L., Su T., Zhang D., Yao D., Li Z. (2023). In vitro simulated fecal fermentation of mixed grains on short-chain fatty acid generation and its metabolized mechanism. Food Res. Int..

[76] Schlörmann W., Keller F., Zetzmann S., Lorkowski S., Dawczynski C., Glei M. (2021). Impact of processing degree on fermentation profile and chemopreventive effects of oat and waxy barley in LT97 colon adenoma cells. Eur. Food Res. Technol..

[77] Paesani C., Sciarini L.S., Moiraghi M., Salvucci E., Prado S., Pérez G.T., Fabi J.P. (2020). Human colonic in vitro fermentation of water-soluble arabinoxylans from hard and soft wheat alters *Bifidobacterium* abundance and short-chain fatty acids concentration. LWT.

[78] Snelders J., Olaerts H., Dornez E., Van de Wiele T., Aura A.-M., Vanhaecke L., Delcour J.A., Courtin C.M. (2014). Structural features and feruloylation modulate the fermentability and evolution of antioxidant properties of arabinoxylanoligosaccharides during in vitro fermentation by human gut derived microbiota. J. Funct. Foods.

[79] Zhang Z., Yang P., Zhao J. (2022). Ferulic acid mediates prebiotic responses of cereal-derived arabinoxylans on host health. Anim. Nutr..

[80] Niemi P., Aura A.-M., Maukonen J., Smeds A.I., Mattila I., Niemelä K., Tamminen T., Faulds C.B., Buchert J., Poutanen K. (2013). Interactions of a lignin-rich fraction from brewer’s spent grain with gut microbiota in vitro. J. Agric. Food Chem..

[81] Kruk M., Lalowski P., Płecha M., Ponder A., Rudzka A., Zielińska D., Trząskowska M. (2025). Prebiotic potential of spent brewery grain—In vitro study. Food Chem..

[82] Gómez B., Míguez B., Veiga A., Parajó J.C., Alonso J.L. (2015). Production, purification, and in vitro evaluation of the prebiotic potential of arabinoxylooligosaccharides from brewer’s spent grain. J. Agric. Food Chem..

[83] Komeno M., Hayamizu H., Fujita K., Ashida H. (2019). Two novel α-L-arabinofuranosidases from *Bifidobacterium longum* subsp. longum belonging to glycoside hydrolase family 43 cooperatively degrade arabinan. Appl. Environ. Microbiol..

[84] Rivière A., Moens F., Selak M., Maes D., Weckx S., De Vuyst L. (2014). The ability of bifidobacteria to degrade arabinoxylan oligosaccharide constituents and derived oligosaccharides is strain dependent. Appl. Environ. Microbiol..

[85] Walton G.E., Lu C., Trogh I., Arnaut F., Gibson G.R. (2012). A randomised, double-blind, placebo controlled cross-over study to determine the gastrointestinal effects of consumption of arabinoxylan-oligosaccharides enriched bread in healthy volunteers. Nutr. J..

[86] Kjølbæk L., Benítez-Páez A., Gómez Del Pulgar E.M., Brahe L.K., Liebisch G., Matysik S., Rampelli S., Vermeiren J., Brigidi P., Larsen L.H. (2020). Arabinoxylan oligosaccharides and polyunsaturated fatty acid effects on gut microbiota and metabolic markers in overweight individuals with signs of metabolic syndrome: A randomized cross-over trial. Clin. Nutr..

[87] Müller M., Hermes G.D.A., Canfora E.E., Holst J.J., Zoetendal E.G., Smidt H., Troost F., Schaap F.G., Damink S.O., Jocken J.W.E. (2020). Effect of wheat bran derived prebiotic supplementation on gastrointestinal transit, gut microbiota, and metabolic health: A randomized controlled trial in healthy adults with a slow gut transit. Gut Microbes.

[88] Schupfer E., Pak S.C., Wang S., Micalos P., Jeffries T., Ooi S.L., Golombick T., Harris G., El-Omar E. (2021). The effects and benefits of arabinoxylans on human gut microbiota—A narrative review. Food Biosci..

[89] Fehlner-Peach H., Magnabosco C., Raghavan V., Scher J.U., Tett A., Cox L.M., Gottsegen C., Watters A., Wiltshire-Gordon J.D., Segata N. (2019). Distinct polysaccharide utilization profiles of human intestinal *Prevotella copri* isolates. Cell Host Microbe.

[90] Vinelli V., Biscotti P., Martini D., Del Bo’ C., Marino M., Meroño T., Nikoloudaki O., Calabrese F.M., Turroni S., Taverniti V. (2022). Effects of dietary fibers on short-chain fatty acids and gut microbiota composition in healthy adults: A systematic review. Nutrients.

[91] Zhang Y., Hu J., Tan H., Zhong Y., Nie S. (2022). *Akkermansia muciniphila,* an important link between dietary fiber and host health. Curr. Opin. Food Sci..

[92] Zhang Z., Wang J., Dang S., Liu X., Zhang Y., Zhang H. (2025). The worldview of *Akkermansia muciniphila*, a bibliometric analysis. Front. Microbiol..

[93] Yang J., Bindels L.B., Segura-Munoz R.R., Martínez I., Walter J., Ramer-Tait A.E., Rose D.J. (2016). Disparate metabolic responses in mice fed a high-fat diet supplemented with maize-derived non-digestible feruloylated oligo- and polysaccharides are linked to changes in the gut microbiota. PLoS ONE.

[94] Song Y., Wu M.-S., Tao G., Lu M.-W., Lin J., Huang J.-Q. (2020). Feruloylated oligosaccharides and ferulic acid alter gut microbiome to alleviate diabetic syndrome. Food Res. Int..

[95] Miquel S., Martín R., Rossi O., Bermúdez-Humarán L.G., Chatel J.M., Sokol H., Thomas M., Wells J.M., Langella P. (2013). *Faecalibacterium prausnitzii* and human intestinal health. Curr. Opin. Microbiol..

[96] Damen B., Verspreet J., Pollet A., Broekaert W.F., Delcour J.A., Courtin C.M. (2012). Prebiotic effects and intestinal fermentation of cereal arabinoxylans and arabinoxylan oligosaccharides in rats depend strongly on their structural properties and joint presence. Mol. Nutr. Food Res..

[97] Jefferson A., Adolphus K. (2019). The effects of intact cereal grain fibers, including wheat bran on the gut microbiota composition of healthy adults: A systematic review. Front. Nutr..

[98] Gonen-Colak B., Turan-Demirci B., Büyüktuncer Z. (2026). Modification of gut microbiome by cereal and pseudocereal consumption: A systematic review. Nutr. Rev..

[99] Méndez-Encinas M.A., Valencia-Rivera D.E., Carvajal-Millan E., Astiazaran-García H., Micard V., Rascón-Chu A. (2021). Fermentation of ferulated arabinoxylan from the maize bioethanol industry. Processes.

[100] Weimer P.J. (2022). Degradation of cellulose and hemicellulose by ruminal microorganisms. Microorganisms.

[101] Tamanai-Shacoori Z., Smida I., Bousarghin L., Loral O., Meuric V., Fong S.B., Bonnaure-Mallet M., Jolivet-Gougeon A. (2017). *Roseburia* spp.: A marker of health?. Future Microbiol..

[102] Vacca M., Celano G., Calabrese F.M., Portincasa P., Gobbetti M., De Angelis M. (2020). The controversial role of human gut *Lachnospiraceae*. Microorganisms.

[103] Heinen G.D., Garzón A.G., Cian R.E., Drago S.R. (2025). Gastrointestinal and colonic bioaccessibility of calcium and ferulic acid from microcapsules made with brewer spent grain arabinoxylans. Int. J. Biol. Macromol..

[104] Liu S., Perez Donado C.E., Rose D.J. (2025). Impact of ferulic and vanillic acids on soluble and insoluble dietary fiber utilization from maize bran by the human gut microbiota. Food Funct..

[105] Zhang X., Xu Z., Liu W., Gao B., Xie J., Chen T., Li E., Li B., Li C. (2025). Strategic alteration of arabinoxylan feruloylation enables selective shaping of the human gut microbiota. Food Hydrocoll..

[106] Pereira G.V., Abdel-Hamid A.M., Dutta S., D’Alessandro-Gabazza C.N., Wefers D., Farris J.A., Bajaj S., Wawrzak Z., Atomi H., Mackie R.I. (2021). Degradation of complex arabinoxylans by human colonic Bacteroidetes. Nat. Commun..

[107] Lynch K.M., Steffen E.J., Arendt E.K. (2016). Brewers’ spent grain: A review with an emphasis on food and health. J. Inst. Brew..

[108] Clavel C., Núñez-Gómez V., Ferrando B.O., Baenas N., Sánchez-Martínez L., González-Barrio R., Santaella M., Periago M.J. (2026). Brewer’s spent gain flour: Chemical composition, functional properties, and influence on gut microbiota. Foods.

[109] Hodgkinson K., El Abbar F., Dobranowski P., Manoogian J., Butcher J., Figeys D., Mack D., Stintzi A. (2023). Butyrate’s role in human health and the current progress towards its clinical application to treat gastrointestinal disease. Clin. Nutr..

[110] Dalile B., Van Oudenhove L., Vervliet B., Verbeke K. (2019). The role of short-chain fatty acids in microbiota–gut–brain communication. Nat. Rev. Gastroenterol. Hepatol..

[111] Naibaho J., Korzeniowska M., Sitanggang A.B., Lu Y., Julianti E. (2024). Brewers’ spent grain as a food ingredient: Techno-processing properties, nutrition, acceptability, and market. Trends Food Sci. Technol..

[112] Czubaszek A., Wojciechowicz-Budzisz A., Spychaj R., Kawa-Rygielska J. (2022). Effect of added brewer’s spent grain on the baking value of flour and the quality of wheat bread. Molecules.

