# Supplementary material for: Prebiotic Potential of Brewer’s Spent Grain Residual Solid After Enzymatic Hydrolysis: Evidence from a Colonic Fermentation Study
Source: Foods. 2026 Jul 3;15(13):2378. doi: 10.3390/foods15132378 (PMC13362525; doi:10.3390/foods15132378)
Supplement: Supplementary file 1 [file foods-15-02378-s001.zip › foods-4392841-supplementary.pdf]

Supplemental material

Table S1. Validation parameters of the HPLC method for the determination of hydroxycinnamic acids.

Hydroxycinnamic acid	Calibration equation	LOD (mg/L)	LOQ (mg/L)	Recovery (%)
Ferulic acid	Y = 48229.0 + 40612.3X	0.9	2.6	99.0–101.8
Sinapic acid	Y = 41553.8 + 10138.3X	1.4	4.5	98.9–102.3
p-Coumaric acid	Y = −1542.8 + 26649.5X	2.2	6.6	95.1–101.6
Chlorogenic acid	Y = 19716.1 + 21477.3X	0.4	1.1	99.4–101.6
Caffeic acid	Y = 112992.0 + 18457.2X	1.1	3.2	99.1–102.2

HPLC, High Performance Liquid Chromatography; LOD, limit of detection; LOQ, limit of quantification.

Table S2. Phenolic profile of the hydrolyzed fractions of brewer's spent grain.

Parameter	HRSF [mg/100 g]	HLF [mg/100 g]	NHBSG [mg/100 g]
**Ferulic acid**	180.84 ± 3.28[a]	43.95 ± 2.22[b]	179.59 ± 3.30[a]
**Sinapic acid**	498.29 ± 16.32[a]	1.57 ± 1.28[b]	472.83 ± 54.78[a]
**p-Coumaric acid**	3.53 ± 0.14[a]	<LOD	2.68 ± 0.60[a]
**Chlorogenic acid**	<LOD	>LOD, <LOQ	>LOD, <LOQ
**Caffeic acid**	<LOD	>LOD, <LOQ	>LOD, <LOQ

HRSF, hydrolyzed residual solid fraction; HLF, hydrolyzed liquid fraction; NHBSG, non-hydrolyzed brewer's spent grain. Values were expressed as mean ± standard deviation (n = 3). Different superscript letters within the same row indicate significant differences according to one-way ANOVA followed by Tukey's test (p < 0.05). LOD, limit of detection; LOQ, limit of quantification.

Table S3. Net total short-chain fatty acid (SCFA) accumulation (mM) over time during *in vitro* colonic fermentation of brewer's spent grain solid samples.

Substrate	Time (h)	ΔSCFA (mM)
**HRSF**	24	0.52 ± 0.52[b]
	48	3.02 ± 2.00[a]
**NHBSG**	24	2.04 ± 0.56[a]
	48	2.96 ± 1.89[a]
**FA**	24	1.93 ± 0.26[a]
	48	1.70 ± 0.37[a]
**FOS**	24	4.32 ± 0.90[b]
	48	6.48 ± 0.63[a]

**NC**	24	2.24 ± 0.82[a]
	48	3.58 ± 1.13[a]

HRSF, hydrolyzed residual solid fraction; NHBSG, non-hydrolyzed brewer's spent grain; FA, ferulic acid; FOS, fructooligosaccharides; NC, negative control. Values were expressed as mean ± standard deviation (n = 3). Different superscript letters within the same substrate indicate significant differences between fermentation times, according to one-way ANOVA followed by Tukey's test ($p < 0.05$).

Table S4. Chemical composition of different batches of brewer's spent grain from the same IPA beer type used in this study.

Parameter	Lot 1* [g/100g]	Lot 2 [g/100g]	Lot 3 [g/100g]
**Moisture**	3.09 ± 0.05	1.73 ± 0.06	0.94 ± 0.01
**Ash**	2.73 ± 0.02	3.02 ± 0.02	2.57 ± 0.03
**Ether extract**	5.98 ± 0.04	6.85 ± 0.24	5.69 ± 0.02
**Protein**	19.85 ± 0.23	18.42 ± 0.08	20.57 ± 0.07
**Total dietary fiber**	48.67	43.78	41.77
**Insoluble fiber**	46.08 ± 0.00	42.28 ± 0.01	38.61 ± 0.00
**Soluble fiber**	2.59 ± 0.00	1.50 ± 0.01	3.16 ± 0.00
**Available carbohydrates**	19.68	26.20	26.20
**Total sugars**	7.91 ± 0.10	11.92 ± 0.14	13.20 ± 0.27

Lot 1* corresponds to the NHBSG sample used in this study. NHBSG, non-hydrolyzed brewer's spent grain. Values are expressed as mean ± standard deviation (n = 3). Available carbohydrates were calculated by difference. Total dietary fiber was calculated as the sum of insoluble and soluble dietary fiber.

Table S5. Percentage distribution of arabinoxylan content in the enzymatically hydrolyzed fractions of brewer's spent grain from two batches of the same IPA beer type used in this study.

Enzymatically hydrolyzed fraction	Lot 1* (%)	Lot 2 (%)
**HLF**	50.84	52.15
**HRSF**	49.16	47.85

Percentage distribution of arabinoxylan was determined in the hydrolyzed liquid fraction (HLF) and the hydrolyzed residual solid fraction (HRSF). Lot 1* corresponds to the NHBSG sample used for enzymatically hydrolyzed in this study. NHBSG, non-hydrolyzed brewer's spent grain. Enzymatic hydrolysis was performed using the commercial β-glucanase Ultraflo® L (Novozymes, Bagsværd, Denmark) under the conditions described by Faulds *et al*. (2002, 2004) [30, 31], with an enzyme loading of 3% (w/w, relative to the raw material weight) at 50 °C.
